# Foot-to-Foot Contact Among Initial Goal-Directed Movements Supports the Prognostic Value of Fidgety Movements in HIE-Cooled Infants

**DOI:** 10.3389/fped.2021.731021

**Published:** 2022-01-05

**Authors:** Fabrizio Ferrari, Luca Bedetti, Natascia Bertoncelli, Maria Federica Roversi, Elisa Della Casa, Isotta Guidotti, Luca Ori, Roberto D'Amico, Lara Valeri, Licia Lugli, Laura Lucaccioni, Alberto Berardi

**Affiliations:** ^1^Neonatal Intensive Care Unit, Women's and Children's Health Department, University Hospital of Modena, Modena, Italy; ^2^PhD Program in Clinical and Experimental Medicine, University of Modena and Reggio Emilia, Modena, Italy; ^3^Unit of Statistics, Department of Diagnostic, Clinical and Public Health Medicine, University of Modena and Reggio Emilia, Modena, Italy; ^4^Pediatric Post-graduate School, University Hospital of Modena and Reggio Emilia, Modena, Italy; ^5^Pediatrics, Women's and Children's Health Department, University Hospital of Modena, Modena, Italy

**Keywords:** fidgety, foot-to-foot, hypoxic ischemia encephalopathy (HIE), therapeutic hypothermia, cerebral palsy

## Abstract

**Background:** Few studies conducted to date have observed general movements in infants affected by hypoxic–ischemic encephalopathy (HIE) who underwent therapeutic hypothermia. We investigated whether foot-to-foot contact (FF) could support the predictive value of fidgety movements (FMs) in infants affected by HIE and treated with brain cooling.

**Methods:** Spontaneous motility was video recorded for 3–5 min at 12 weeks post-term age in 58 full-term newborn infants affected by perinatal asphyxia who were cooled due to moderate to severe HIE. FF and FMs were blindly scored by three independent observers. At 24 months, each patient underwent a neurological examination by Amiel-Tison and Grenier.

**Results:** At 24 months, 47 infants had developed typically at neurological examination, eight had developed mild motor impairment, and three developed cerebral palsy (CP). At 12 weeks, 34 (58.6%) infants had shown normal FMs, four of whom developed mild motor impairment. Twenty-four infants (41.4%) exhibited abnormal or no FMs, four of whom developed mild motor impairment and three developed CP. FF was present in 20 infants (34.5%), two of whom developed mild motor impairment. FF was absent in 38 infants (65.5%), six of whom developed mild motor impairment and three developed CP. Both FMs and FF, considered separately, were 100% sensitive for predicting CP at 24 months, but only 61 and 36%, respectively, were specific. Summing the two patterns together, the specificity increases to 73%, considering only CP as an abnormal outcome, and increases to 74% when considering CP plus mild motor impairment. Unexpectedly, fidgety movements were absent in 24 infants with typical motor outcomes, 17 of whom showed a typical motor outcome.

**Conclusions:** FF is already part of motor repertoire at 12 weeks and allows a comparison of spontaneous non-voluntary movements (FMs) to pre-voluntary movements (FF). FF supports FMs for both sensitivity and specificity. A second video recording at 16–18 weeks, when pedipulation is present in healthy infants, is suggested: it may better define the presence or absence of goal-directed motility.

## Introduction

Perinatal asphyxia remains one of the most important causes of death and neurological sequelae, being the cause of 6–23% of cerebral palsy (CP) in childhood. Moderate or severe hypoxic encephalopathy has a mortality rate of 10–60%, and 10–30% of infants affected by hypoxic–ischemic encephalopathy (HIE) develop CP (1), while 10–30% of infants develop mild motor impairment, cognitive, and behavioral problems, and only 50% show typical development. In recent decades, clinicians and researchers have stressed the importance of early diagnosis of perinatal brain injury and the correct prognosis for individual infants affected by perinatal asphyxia.

General movement assessment (GMA), neurological examination, and MRI represent the most powerful tools for the early diagnosis of CP. Currently, early diagnosis and prognosis of brain dysfunction, of CP in particular, is mandatory to select those infants that warrant a strict neurological follow-up and an early intervention strategy. A large panel of experts has recently recognized “the importance of prompt referral to diagnostic-specific early intervention to optimize infant motor and cognitive plasticity, prevent secondary complications, and enhance caregivers' actions” (2).

The diagnostic and prognostic power of GMs and of fidgety movements in particular has been confirmed by a number of studies (3–12) and by three systematic meta-analyses (2, 13, 14). The introduction of therapeutic hypothermia, however, has markedly changed the outcome for newborns suffering from HIE; therefore, the predictive value of GMs has to be recalibrated from the precooling to the cooling era. In non-cooled HIE infants observed in the pre-cooling phase, we previously found that severe MRI abnormalities of basal ganglia and thalami were landmarks of an acute severe global hypoxic–ischemic event, and a severe lesion of basal ganglia and thalami was highly correlated with either abnormal GMs [absent/abnormal fidgety movements (FMs), cramped synchronized movements] or CP in HIE infants (3). More recently, we investigated whether the combination of GMs with other motor and postural patterns pointing to goal-directed antigravity movements support GMs for the early diagnosis of CP (15). Eleven concomitant motor and postural patterns in preterm infants assessed longitudinally through preterm, term, and fidgety age in combination with GMs were associated with CP for all 11 pathological patterns at fidgety age. Moreover, lower limb movements toward the midline [as part of movements toward the midline (MTM), such as foot-to-foot, hand-to-hand, hand-to-face, hand-to-mouth, and hand-to-trunk contact] were the second most powerful pattern associated with CP after monotonous and stereotyped limb movements.

Starting from these premises and considering that 12–14 weeks post-term age (PTA) is a key age in development (1, 16), as fidgety movements are at their best expression (17) and the age-adequate motor repertoire is emerging as a new paradigm reflecting goal-directed motility, we considered foot-to-foot contact (FF) in 58 HIE-cooled full-term infants whose motility was video-recorded at 12 weeks. We addressed two specific questions: Is the 12th week PTA suitable for comparing spontaneous motility (and fidgety movements in particular) to goal-directed FF? Does FF contact support the prediction of later motor outcomes performed on the basis of FMs?

## Materials and Methods

We performed a retrospective observational study. The study group comprised term infants born at or referred to Modena University Hospital's NICU with a diagnosis of hypoxic–ischemic asphyxia, who underwent therapeutic hypothermia (in accordance with Italian Guidelines) (18) from March 1, 2009, to July 31, 2017. Eligible patients were scheduled to complete neurological follow-up at 24 months of age.

Data collected regarded obstetric and neonatal history (type of delivery, sex, birth weight, Apgar score, and grade of encephalopathy), assessment of general movements (GMs), and of lower limb movements toward/across midline (FF) at fidgety age (from 50 to 54 weeks PTA), and motor outcome at 24 months of age. GMs and FF were evaluated independently by three expert neonatologists and a physical therapist, all blinded to neurological outcome. Video recordings of 3–5 min duration, registered in accordance with Prechtl's method (16) were reviewed. Inter-observer agreement was noted, and in case of disagreement, the team came to a final score by reviewing the videos. FMs, seen between 46 and 60 weeks PTA, are small-amplitude movements of moderate speed and variable acceleration of the neck, trunk, and limbs in all directions and continual in the awake infant except during crying. Absence of fidgety movements consists of the total absence of fidgety; sporadic fidgety movements are those present for <50% of observational time; and abnormal fidgety movements are fidgety-like movements with moderately or greatly exaggerated amplitude, speed, and jerkiness. Fidgety movements were scored as present (F+) or absent (if absent, sporadic, or abnormal). FF consists of lifting legs off the mattress with subsequent movements toward or across the midline and sliding or grabbing movements of the feet. Five subtypes of this motor pattern can be recognized: plant-to-plant contact, plant to the tibial margin of the contralateral foot, plant to the dorsal part of the contralateral foot, feet crossing, and pedipulation—the last and most complex type of FF, usually appearing from 52 to 54 weeks PTA onward, and characterized by variable and prolonged indulging in FF. From its first appearance, pedipulation increases in frequency, reaching a plateau after 20 weeks PTA when fidgety movements have already disappeared. Neurological outcome was scored by evaluating a neurologic examination performed at 24 months of age, in accordance with Amiel-Tison and Grenier protocols (19). Motor outcome was classified as typical, mild motor impairment (clumsiness and/or poor balance, without CP), or as CP (permanent but not unchanging disorder of movement or posture or both and of motor function). Developmental outcome was evaluated using the Griffiths Mental Development Scale (GMDS-II) assessment, but these are not presented in this study. The study was approved by the local ethics committee (CE 405/17).

The characteristics of infants, GMs, FF, and neurologic outcomes were summarized using descriptive statistics. Continuous variables were expressed as mean ± standard deviation, whereas categorical variables were expressed as frequencies. Sensitivity and specificity of FMs, FF, and FMs + FF were calculated with respect to the motor outcome at 24 months.

## Results

### Outcome of the Study Group

Perinatal clinical characteristics of infants enrolled are shown in Table 1. Forty-seven infants had typical motor outcome (no neurologic signs at the neurological exam), eight showed mild motor impairment, and three suffered from CP at 24 months of age. Of the three with CP, two were affected by spastic hemiplegia and one by a combination of spastic and dystonic tetraplegia.

**Table 1:** Perinatal clinical characteristics of HIE-cooled infants enrolled.

		***N* = 58**
Weight (percentile)	Mean ± SD (range)	61.7 ± 24.8 (6–100)
Apgar 10'	Mean ± SD (range)	5 ± 1.6 (1–9)
Gestational age (weeks)	Mean ± SD (range)	40 ± 1.2 (37–42)
pH Blood gases analysis	Mean ± SD (range)	6.0 ± 0.2 (6.7–7.37)
BE Blood gases analysis	Mean ± SD (range)	−17.2 ± 6 (−31–2)
Sex (male)	*N* (%)	33 (50.8%)
Inborn	*N* (%)	26 (44.8%)
**Delivery**		
Vaginal	*N* (%)	40 (68.9%)
Urgent Cesarean Section		18 (31.1%)
Sentinel events^*^	*N* (%)	5 (8.6%)
CTG alterations^**^	*N* (%)	31 (53.4%)
**HIE grade**		
II	*N* (%)	35 (60%)
III		23 (40%)
**Ethnicity**		
Caucasian	*N* (%)	46 (79.3%)
Not Caucasian		12 (20.7%)

*HIE, hypoxic–ischemic encephalopathy*.

* *Placental abruption, uterine rupture, and umbilical cord prolapse*.

** *CTG, cardiotocography*.

### Presence of Foot-to-Foot Contact

In 20 of the 58 infants, we scored FF as present. As our interest was single subtypes of FF (plant to plant, plant to tibial margin, plant to the dorsal part of contralateral foot, and finally feet crossing), we found that seven infants displayed only one type (mainly plant to plant), eight displayed two types (predominantly plant to plant and/or plant to the tibial margin contact, and/or plant to the dorsal side of the contralateral foot), three displayed three types, and two displayed all four types. None displayed pedipulation.

### Correlation Between Foot-to-Foot Contact and FMs

**Figure 1:**
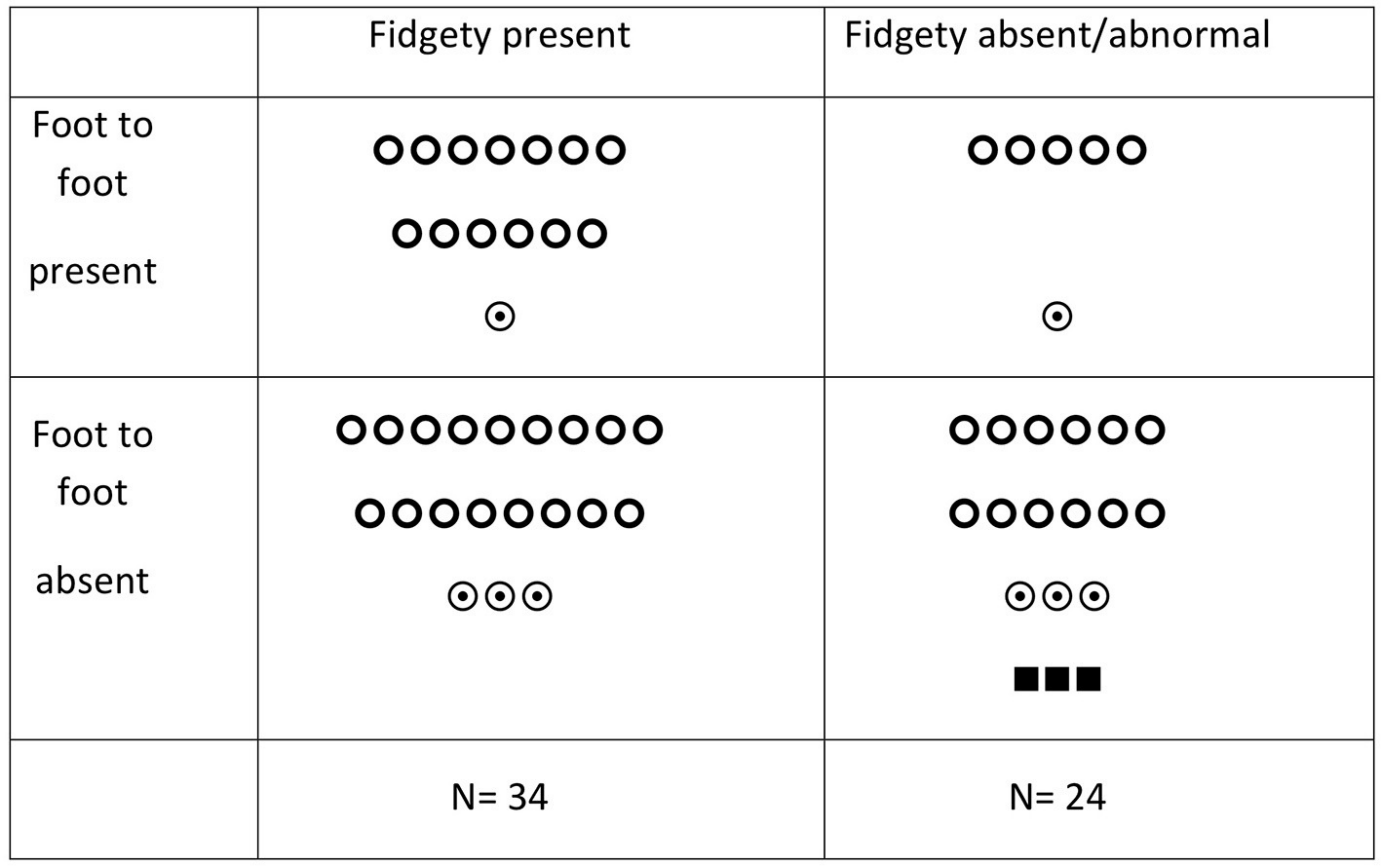
Foot-to-foot contact, fidgety movements, and motor outcome in HIE-cooled infants. 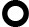 = typical outcome; 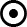 = mild motor impairment; ■ = CP.

Figure 1 and Table 2 show the association between FF and FMs in relation to the outcome at 24 months. Twenty infants (34.5%) showed FF present: 18 were typical and two developed mild motor impairment at 24 months. FF was absent in 38 infants (65.5%); three of them developed CP and six developed mild motor impairment. The remaining had typical motor outcomes. Fidgety movements were present in 34 infants (58.6%): 30 of them had a typical outcome, four developed mild motor impairment, and none developed CP. Twenty-four infants (41.4%) showed the absence of FMs: three of them developed CP and four developed mild motor impairment.

**Table 2:** Combination of foot-to-foot contact and fidgety movements and risk of developing cerebral palsy, mild motor impairment, or both.

**Combinations**	***N***	**Cerebral palsy**	**Mild motor impairment**	**Cerebral palsy or**	**Typical outcome**	Risk of developing CP or mild motor impairment^*^	
		***N* (%)**	***N* (%)**	**mild motor impairment *N* (%)**	***N* (%)**	
						**% (95% CI)**	**OR (95% CI)**
Fidgety present, FF present	14	0 (0)	1 (7.1)	1 (7.1)	13 (92.9)	7% (1.2; 31.5)	Reference
Fidgety present, FF absent	20	0 (0)	3 (15)	3 (15)	17 (85)	15% (5.2; 36.0)	2.3 (0.21; 24.7)
Fidgety absent, FF present	6	0 (0)	1 (16.7)	1 (16.7)	5 (83.3)	16% (3.0; 56.4)	2.6 (0.13; 50.0)
Fidgety absent, FF absent	18	3 (16.7)	3 (16.7)	6 (33.4)	12 (66.6)	33% (16.3; 56.3)	6.5 (0.68; 62.1)
Total	58	3 (5.2)	8 (13.8)	11 (19.0)	47 (81)	-	-

*The table shows that the risk of developing CP or mild motor impairment increases when foot-to-foot contact or fidgety is absent, and that it is maximum when both (foot-to-foot contact and fidgety) are absent*.

* *Risk is ratio expressed as percentage*.

When the combination of FMs and FF were considered in relation to an abnormal outcome (see Table 2), four groups were identified:

1. FM+ and presence of FF were seen in 14 infants; only one developed mild motor impairment, all the others were typical; there were no cases of CP.
2. FM+ and absence of FF were seen in 20 infants; three developed mild motor impairment and 17 had typical outcome.
3. FM– and absence of FF: of the 18 infants, 12 were typical, three developed CP, and three developed mild motor impairment.
4. FM– and presence of FF: of the six infants, five were typical and one developed mild motor impairment.

These data indicate that when FMs and/or FF are present, one or both, the large majority (87.5%) of infants will be typical, only a minority (12.5%) will have the risk of developing mild motor impairment, and none will develop CP. The highest risk of developmental problems is related to the absence of both FMs and FF—of the 18 infants with this combination, three developed CP and three developed mild motor impairment. The other 12 were typical. Finally, of the six infants with both FMs and FF present, only one developed mild motor impairment; the other five were typical. Due to the small number of infants with abnormal outcomes, no statistical analysis was appropriate.

### Sensitivity and Specificity of FMs, Foot-to-Foot Contact, and Motor Pattern Values in Relation to Outcomes at 24 Months

Sensitivity of FMs to predict CP was 100% and specificity was 61%; sensitivity to predict CP + mild motor impairment was 63% and specificity was 63%.

Sensitivity of FF to predict CP was 100% with 36% specificity; sensitivity to predict CP + mild motor impairment was 81% with 38% specificity.

Adding the values of sensitivity and specificity of both FF and FMs, the sensitivity to predict CP was 100% with 73% specificity, and sensitivity to predict CP + mild motor impairment was 54% with 74% specificity.

The sensitivity and specificity of single patterns differ greatly according to CP alone or CP and mild motor impairment together. In fact, if we consider the power to predict CP alone, the sensitivity is 100% for FMs, both for FMs and for FF. On the other hand, if we consider CP + mild motor impairment together as an abnormal outcome, the sensitivity goes down from 100 to 63, 81, and 54% for FMs, FF, and the sum of the two patterns, respectively. Specificity, by contrast, mildly increases in the three categories: from 61 to 63% for FMs, 36 to 38% for FF, and 73 to 74% for the two patterns together.

## Discussion

Our study explored the relationships at 3 months PTA between GMs and FF. It is worth reviewing the nature (voluntary vs. involuntary movements) and the ontogeny of the two motor patterns.

GMs are endogenously generated movements, observed from the early phase of embryofetal life (8–10 weeks post-conception), that maintain their basic features (fluency, complexity, and variability) throughout the whole pregnancy or, in the case of preterm birth, during the preterm and term period. At 6–10 weeks PTA, a new feature, FMs—first sporadic and intermittent, later continual—emerges and substitutes the writhing GMs. Fidgety movements consist of small movements of moderate speed and variable acceleration in all directions of single body parts (i.e., neck, trunk, and limbs) that are continual in the awake infant. From 12 to 16 weeks PTA, FMs become smaller in amplitude and more continual than before; this time period is considered the golden age for fidgety movements that tend to diminish and finally disappear by 18–20 weeks (17).

Similar to GMs, MTMs (and FF in particular) are largely present during fetal life. Thanks to the limited room available *in utero*, the flexed body posture promotes the upper and lower limbs continually reaching for the head, trunk, and contralateral parts of the body. Soon after birth and during the first 4 weeks of life, MTMs are short in duration (<1 s) and occasionally due to the persistence of the *in utero* posture. In the period 4 to 8 weeks after birth, MTM decreases in frequency as the limbs tend to move away from the midline and the force of gravity limits vertical movements. Only by 9 weeks does the MTM tend to increase again, and by 12 weeks, most infants show a constant increase in the number and complexity of MTM; from 16 weeks onward, all infants show manipulation and pedipulation. The insistent play of limbs with other parts of the body (such as hands reaching for the feet and bringing them to the mouth) clearly expresses the voluntary character of these movements (1, 2, 13, 14, 16, 20–23). It is likely that FF at 12 weeks PTA is just a preliminary form of voluntary movements that precedes manipulation and pedipulation. This age corresponds to the emergence of MTM and goal-directed movements that allow the baby to reach for and grasp objects. Differing from GMs that are thought to be generated by the central pattern generators of the brain stem and modulated by cortical and subcortical brain structures, MTM would be generated by the cortical motor neurons present in the precentral fissure, the site where voluntary movements are planned and generated after having received sensory inputs from the post-rolandic fissure and all the ascending sensory input. The main aim of this study was to explore the relationship between spontaneous motility (FM) and goal-directed FF in relation to the outcome.

The data indicate that when FMs and FF are both present, the great majority of infants will be typical, and only a minority will develop mild motor impairment; none will develop CP. Conversely, the highest risk for developmental problems is related to the combined absence of both FMs and FF. Of the 18 infants with this combination, three developed CP and three developed mild motor impairment; the other 12 were typical. Due to the small number of infants with abnormal outcomes, no statistical analysis was appropriate.

Looking at this issue from the perspective of outcome, it can be said that both FMs and FF can independently select the infants at risk for CP, whereas neither FMs nor FF can select infants at risk for mild motor impairment.

Twenty of the asphyxiated infants in our study showed the presence of FF at 12 weeks. It is encouraging that all but two (who later developed mild motor impairment) of the 20 HIE-cooled infants with FF present had a typical outcome, indicating that FF at 12 weeks PTA seems to be a sign of recovery from a hypoxic–ischemic insult. In addition to the aforementioned features, another aspect that works in favor of considering FF as a possible prognostic indicator is in regard to the variety of foot-to-foot subtypes observed in the 20 infants: four subtypes were observed in this sample, namely, plant-to-plant contact, plant to the tibial margin of contralateral foot, plant to the dorsal part of the contralateral foot, and feet crossing. Pedipulation was not observed in the study group, as 12 weeks is too early for this pattern. Lucaccioni et al. (24) found that pedipulation appears later; it was present in 68% of term infants at 14–15 weeks PTA and in 95% of the same group at 20–21 weeks PTA. From the same study (24), we learned that plant-to-plant contact is the first (10–12 weeks PTA) and pedipulation is the last (16–17 weeks PTA) pattern to appear. Another interesting feature emerging from this study was that all 20 infants displayed two to four of the five subtypes within the same sessions of video observation. This means that FF has variable modes of expression. Variable modes of processing a goal-directed movement, according to Bert Touwen's theory (25), is indicative of integrity of the brain structures responsible for MTM patterns. We suggest that the predictive value of FF may be confirmed by adding other evaluations to the 12-week recordings to include 16- to 18-week recordings. This could add information regarding the clinical significance and occurrence of the different subtypes of FF in this age group.

In conclusion, the answer to the first research question seems positive—FF can already be recognized at 12 weeks, consists of a variety of different FF modes, and is synergic to FMs to identify infants at higher risk. Conversely, the lack of both FF and FMs could be due to the effect of hypoxic–ischemic injury that may delay or suppress the emergence of this pattern. A second video at 16–18 weeks is highly recommended.

Regarding the second research question (Do FF movements support the prognostic value of FMs?), the two motor patterns, FF and FMs, were observed at a mean age of 12 weeks PTA, which is the optimal time for observing FMs but too early for observing the whole range of FF and the presence of the more mature pattern, pedipulation. Nevertheless, the answer is positive because both patterns have similar predictive power for CP with high sensitivity (100%) but low specificity (61% for FMs and 36% for FF). Indeed, if we consider the two patterns together, sensitivity remains 100%, and specificity increases to 73%. This upward shift in specificity is important. We feel that clinicians in charge of early diagnostic and prognostic assessment have a new tool in their hands. At 3 months, the presence of FMs and FF already attests to early normalization of brain dysfunction caused by moderate to severe HIE, whereas the absence of one or both of them reveals that brain dysfunction may still be present but does not exclude the possibility of later normalization. Whether FMs are present or absent, the presence of FF is an extra marker pointing to typical development of HIE-cooled infants. In the presence of FF, clinicians and parents may be reassured, despite the absence of FMs.

Finally, this study disclosed new unexpected findings, probably due to therapeutic hypothermia having significantly changed the outcome for newborns suffering from HIE and to the predictive power of markers of poor outcomes witnessed in the precooling phase (26). Analogous to the change in predictive value of the 10-min Apgar score (less predictive in the cooling era than in the precooling era) and clinical assessment of HIE in electing hypothermia (less predictive of outcome than in non-cooled infants), we found that the absence of fidgety had lower predictive power with respect to evaluations made prior to the cooling era. The data from our study showed poor predictive value (61%) of FMs, whereas previous studies showed a high sensitivity for CP in the absence of FMs. A number of studies (3, 27–29) found that the absence of FMs was a strong marker for CP and/or mental and cognitive defects. We observed many infants [(12), i.e., 20%] who exhibited absences of both FMs and FF but had typical outcomes. The high sensitivity of FMs at 3 months is linked to the fact that recovering from HIE was either incomplete or absent, because in full-term infants, recovery takes place early—during the first 3 months of life. This did not happen in these 12 infants who recovered to typical motor behavior only after the fidgety period, that is, at 6 months in seven infants, at 12 months in four of them, and at 24 months in one infant. Another way to interpret this late normalization is to attribute it to a specific effect of brain cooling, which is the ability to reduce brain injury during the acute stage of the HIE and produce brain recovery from the brain insult in the long run, not in the short run. What happens in the central nervous system after this acute phase of brain injury is largely unknown. Whatever the pathophysiology of these motor phenomena, this late normalization must be kept in mind as a possible evolution of moderate to severe HIE treated with brain cooling. Late normalization may be due to support from the staff in charge of follow-up and the early intervention team, and of course of the parents. The presence of either of the two motor patterns led to the typical outcome in the near totality of cases. CP cannot be excluded only when both patterns are absent.

### Limitations of the Study

The most important limitation of the study is linked to the follow-up protocol used in our unit, which calls for the first clinical post-discharge check at 3 months of age. Three months is fine for FMs, as it corresponds to full-blown fidgety movements, but it is too early for MTM and FF in particular, as they reach their best development (manipulation and pedipulation) only from 16 weeks onward. The 12-week video recordings present a picture of FF at its beginning, not as it reaches its plateau. Probably a second video at a later age (e.g., 16–18 weeks PTA), unavailable in our cases, could detect more complex FF, such as pedipulation, thereby increasing sensitivity and specificity values and identifying a larger number of those infants who normalize their movements later than at 12 weeks. A second limitation is that we focused on FF, but this neurological pattern has not been validated as an independent item of neurological assessment. FF has been included in several studies dealing with age-adequate motor repertoire (23, 24), but not as a single motor pattern as we propose in the present study. New and larger studies may confirm the prognostic value of this item as part of the motor repertoire.

In conclusion, this study shows that FF is suitable for comparison with FMs and supports the sensitivity and specificity of fidgety movements. FF is a simple, easy-to-score motor pattern, and it presages the emerging goal-directed movements. It consists of various FF motions, from plant-to-plant contact to pedipulation; the more variable the modes of FF the better, in terms of brain integrity. A suggestion for future studies is to obtain a second video recording at around 16–20 weeks when FF and pedipulation in particular are fully expressed.

## Data Availability Statement

The raw data supporting the conclusions of this article will be made available by the authors, without undue reservation.

## Ethics Statement

The studies involving human participants were reviewed and approved by Comitato Etico Area Vasta Emilia Nord (protocol CE 405/17). Written informed consent to participate in this study was provided by the participants' legal guardian/next of kin.

## Author Contributions

FF, LB, and LLuc conceptualized and designed the study, coordinated and supervised data collection, drafted the initial manuscript, and reviewed and revised the manuscript. NB, LO, LV, and IG collected data and drafted the initial manuscript. LLug, MR, EDC, RD'A, and AB critically analyzed and interpreted data and critically reviewed the manuscript. All authors approved the final manuscript as submitted and agree to be accountable for all aspects of the work.

## Conflict of Interest

The authors declare that the research was conducted in the absence of any commercial or financial relationships that could be construed as a potential conflict of interest.

## Publisher's Note

All claims expressed in this article are solely those of the authors and do not necessarily represent those of their affiliated organizations, or those of the publisher, the editors and the reviewers. Any product that may be evaluated in this article, or claim that may be made by its manufacturer, is not guaranteed or endorsed by the publisher.
